# Towards an Implantable, Low Flow Micropump That Uses No Power in the Blocked-Flow State

**DOI:** 10.3390/mi7060099

**Published:** 2016-06-01

**Authors:** Dean G. Johnson, David A. Borkholder

**Affiliations:** Rochester Institute of Technology, Microsystems Engineering, Rochester, NY 14623, USA; dgj2607@rit.edu

**Keywords:** direct write, gallium, Hybrid MEMs, implantable, integration, micropump, microsystems, peristaltic, and phase-change

## Abstract

Low flow rate micropumps play an increasingly important role in drug therapy research. Infusions to small biological structures and lab-on-a-chip applications require ultra-low flow rates and will benefit from the ability to expend no power in the blocked-flow state. Here we present a planar micropump based on gallium phase-change actuation that leverages expansion during solidification to occlude the flow channel in the off-power state. The presented four chamber peristaltic micropump was fabricated with a combination of Micro Electro Mechanical System (MEMS) techniques and additive manufacturing direct write technologies. The device is 7 mm × 13 mm × 1 mm (<100 mm^3^) with the flow channel and exterior coated with biocompatible Parylene-C, critical for implantable applications. Controllable pump rates from 18 to 104 nL/min were demonstrated, with 11.1 ± 0.35 nL pumped per actuation at an efficiency of 11 mJ/nL. The normally-closed state of the gallium actuator prevents flow and diffusion between the pump and the biological system or lab-on-a-chip, without consuming power. This is especially important for implanted applications with periodic drug delivery regimens.

## 1. Introduction

A low flow rate, low power, biocompatible micropump capable of periodic flow is critical for drug delivery to small spaces and in small rodent models, such as with ocular and cochlear infusions, and fluid sensitive areas which can be damaged by over perfusion, such as with neural tissue [1]. Small pumps capable of pumping small volumes of fluid are also needed in a number of applications including portable diagnostics, particle and cell sorting, and cytometry. Resistance to backflow and fluid traveling from target areas is also a requirement for these applications, as studies have shown that backflow from the biological systems into the pump is problematic for infusion rates below 500 nL/min [2,3].

Pumps for ocular infusions include both research and commercial devices. Research devices used for rabbits have been designed to pump up to 1.5 µL/min [4], 5 µL/min to 20 µL/min [5], and 7.0 µL/min in enucleated porcine eyes [6]. Commercial devices include the ALZET pump (ALZET Osmotic Pumps, Cupertino, CA, USA), which has been used for transscleral delivery to choroid and retina of rabbits at 133 nL/min [7]. Pump rates for cochlear infusions in small rodent models require even lower flow rates than are currently available due to smaller fluid volume and pressure sensitive structures. In the mouse model, infusions were performed at 17 nL/min [8], 16 nL/min to 32 nL/min [9], and up to 50 nL/min with posterior semicircular canal canalostomy [10].

Point of care diagnostic systems often rely on small sample sizes that necessitate low flow rate pumps [11]. These types of devices have been designed with flow rates of up to 100 nL/min [12,13,14]. For example, micro total analysis systems (µTAS) perform sample manipulation on small fluid volumes less than 1 nL [15], including sample delivery to perform mixing, reaction, and analysis. 

In drug delivery applications diffusion often plays a role in delivery, but can also result in undesired diffusion mediated transport during periods when the pump is inactive. Therefore, a means of blocking the flow to isolate the drug reservoir from the biological system is needed for implantable systems, which can be difficult in these power-constrained applications. While none of the research pumps presented above achieve blocked flow, commercial products such as the SynchroMed II from Medtronic (Minneapolis, MN, USA) pump as low as 0.6 µL/min and do provide flow blockage, but only by powering the pump in the off position. The iPRECIO pumps (ALZET Osmotic Pumps, Cupertino, CA, USA) are essentially miniaturized versions of a standard peristaltic pump using pins to compress tubing in place of the standard roller mechanism and provide flow rates down to 60 µL/min. While both of these are programmable and provide blocked flow, the minimum flow rates are much larger than those required for implantable small rodent and μTAS systems. Additionally, they require power to maintain the blocked-flow condition. There is a need for ultra-low flow rate implantable pumps that provide the flexibility of the iPRECIO pump, with the added benefit of blocked flow in the unpowered state. 

**Figure 1:**
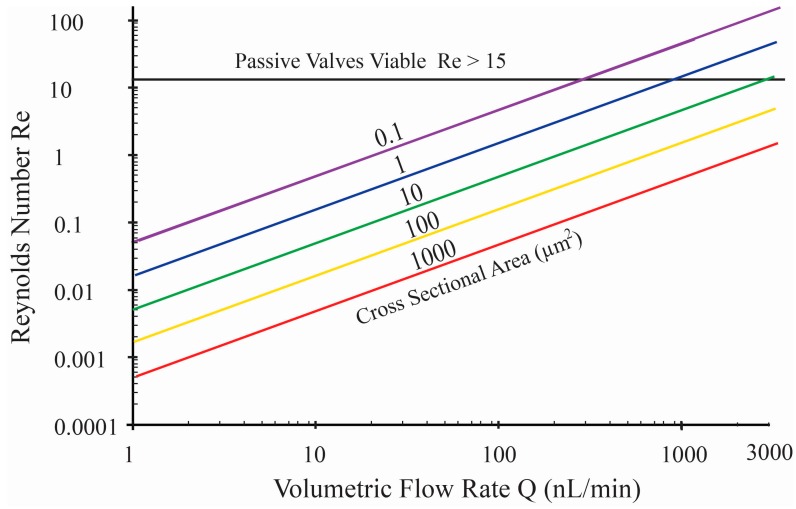
Family of curves showing Reynolds number *vs.* flow rate in rectangular channels for various flow channel cross sectional sizes. Passive valves are ineffective in the laminar flow regime requiring active valves for robust flow rectification.

Valves can be active such as a diaphragm actuated with external force, or passive as in the case of a flap check valve where pressure of the pumped fluid opens and closes the valve. This type of valve requires a minimum volume to deform the valve flap sufficiently before fluid begins to flow. Check valves have been reported with dimensions ranging from 70,000 μm^2^ [16] to 2 mm^2^ [17], which are too large for low flow rate micropumps. Passive valves also include fixed geometry valves, which rely on the fluid mechanics within the channel to favor flow in one direction over another, resulting in flow rectification. However, in the laminar flow regime, hydrodynamics must be considered when evaluating the utility of passive valves [18,19,20]. These passive valves rely on the hydrodynamics of the system to produce a valve effect, so the Reynolds number (*Re*), the ratio of the inertial to viscous forces, defines valve performance. Systems with low *Re* require careful consideration in the design phase to ensure sufficient flow rectification to enable pumping. The critical number below which flow is completely laminar is *Re* = 15; below this value, valves relying on pressure loss coefficients (e.g., fixed geometry valves) have been shown not to function [21], and check valves exhibit efficiencies below 12% [22]. Figure 1 shows a graph of *Re* compared to volumetric flow rates for various channel cross sectional areas (*A*_CS_). For microfluidic cross-sectional areas that can be easily fabricated (e.g., *A*_CS_ > 10 μm^2^), flows remain completely laminar for flow rates below 3000 nL/min, making passive valves impractical. This remains true even for peristaltic pumps with pulsatile flow, as long as the instantaneous flow rate associated with displacement actuation remains below this critical value. Hence, in this work, active valves were pursued.

There are numerous actuation mechanisms that can be used with active valves including electrostatic, piezoelectric, electromagnetic, electro-osmotic, electrolysis, magneto-hydrodynamic, thermodynamic, continuous electro-wetting, shape memory material, and phase-change material [23,24,25]. For low power consumption in a programmable micropump, it is important to use normally closed valves to restrict flow without expending energy. Normally closed active valves can be achieved with shape memory alloys, however these generally require relatively high temperatures to deform (e.g., 75 **°**C [26]) that may be incompatible with the biological system or degrade the drug being pumped. An alternative is phase-change materials that expand when they solidify, and shrink during melting. Gallium is a suitable material for this application as it has a melting point sufficiently low to prevent damage to the biological system, and it undergoes orthorhombic crystallization as it cools causing the material to expand as the crystals are formed. In this paper we describe a gallium actuator in a fully integrated micropump with the underlying chamber-based peristaltic pump providing the active valves required for zero-power blocked flow. The pump was created with a hybrid Micro Electro Mechanical System (MEMS) approach including the integration of biocompatible membranes and channels [27] with the Ga actuator using additive manufacturing, direct write techniques. The performance of the fully integrated pump was characterized including chamber volumes, pump rates, and pump efficiency.

## 2. Design

**Figure 2:**
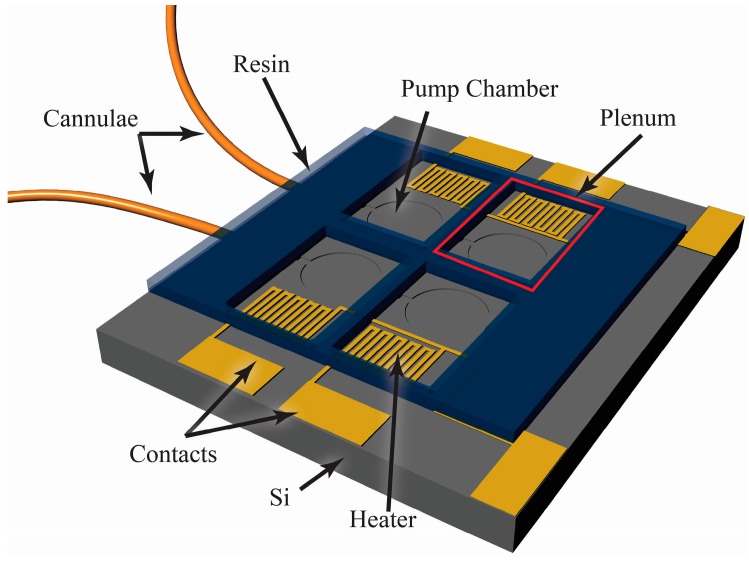
Conceptual illustration of a four-chamber peristaltic pump (6.4 mm × 4.4 mm × 1 mm) for use with phase-change actuation, coupled to small diameter capillary tubing.

The foundation of the peristaltic pump is a silicon-based MEMS device, which includes fluidic channels, in-plane interconnects, pump chambers, and an etched aluminum film creating heaters and contact pads as illustrated in Figure 2. Four phase-change actuators were used in this pump to directionally motivate the fluid peristaltically by transferring momentum to the underlying Tetraethyl Orthosilicate (TEOS)/Parylene-C diaphragms. Parylene-C was chosen as it is a non-reacting, non-water-adsorbing, biocompatible material that can be deposited in a pseudo-conformal process [28].

Parylene-C has been shown to be biocompatible with *in vitro* studies including live/dead staining and cell viability [29], and *in vivo* tests for inflammation [30] and tissue morphologic changes [31,32]. Material structural stability when implanted has been confirmed with long-term *in vivo* studies [33]. The demonstrated biocompatibility, biostability, and the ability to encapsulate implant devices [34] make Parylene-C an ideal coating for implantation. The presented device used Parylene in four ways, first to form the pump chamber diaphragms, second to provide a biocompatible flow path, third to capture small diameter tubing for fluid delivery, and fourth to encapsulate the entire device in a stable, biocompatible coating.

**Figure 3:**
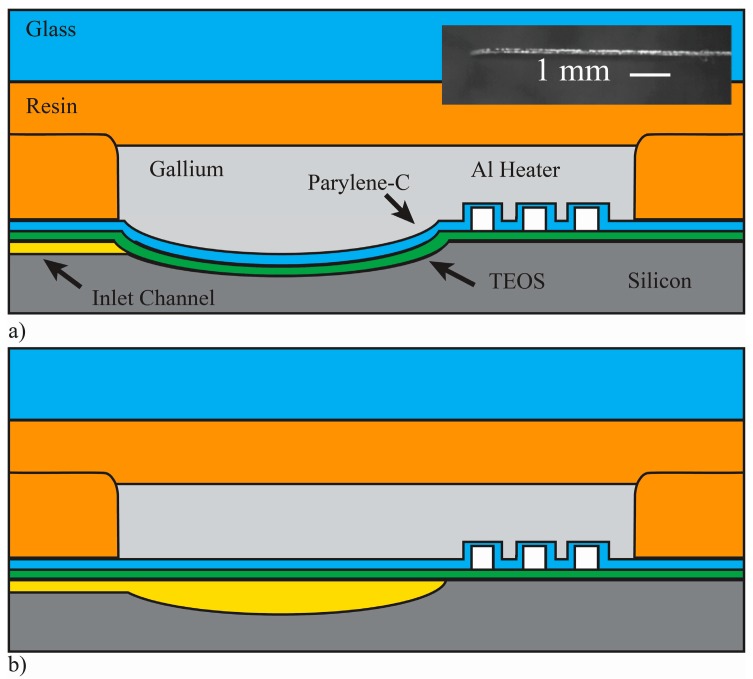
Gallium-based phase change actuator in the solidified (closed) state (**a**); and liquified (open) state (**b**). *Inset*: micro-ingot of Ga used for length-based resolution of sub-mg quantities.

Aluminum resisters were used to transfer actuation energy in the form of heat to the gallium, which resides in plena formed via a direct-write method over the pump chambers. The rigid plena provide a constant volume above each diaphragm, such that expansion and contraction of the gallium results in actuation of the diaphragm. As the gallium is heated to its melting point, it loses its orthorhombic crystal structure and collapses, resulting in a reduction in volume and upward movement of the flexible diaphragm pulling fluid into the underlying pump chamber. As the gallium cools and freezes, expansion forces the diaphragm down, pushing fluid out of the chamber and effectively closing the flow path. The actuation mechanism and pump structure are illustrated in Figure 3.

The compression ratio of the pump chambers needs to be great enough to overcome back pressure from the downstream system (e.g., biological system). For flow rates appropriate for implantable systems in small rodent models, peristaltic pump chambers need to be tens of microns deep and hundreds of microns in diameter to provide sufficient compression ratio (diaphragm displacement volume to total chamber volume) to deliver fluid against back pressure [23]. With the current fabrication process, chambers of up to 1000 µm can easily be fabricated. Channels, the same depth as the chambers, can easily be fabricated as narrow as 10 µm and as wide as the pump chambers. For the current work, channels 100 µm wide have been used providing a channel cross-sectional area of 1500 μm^2^ and completely laminar flow for flow rates up to 60 μL/min. Chamber and membrane designs ranged from 500 to 800 µm diameter, 15 µm deep with 3 to 4.5 µm of deposited Parylene C (PCPX) deposited over 1 μm of TEOS. 

The maximum back-pressure against which the gallium actuators can work was estimated from the bulk modulus of Ga (56.9 GPa). Gallium expands 3.1% when it solidifies, providing a theoretical, maximum pressure of 1.76 GPa to the diaphragm and underlying fluid. This is more than ten times the expansion pressure provided by a paraffin wax actuator with a bulk modulus of 1.66 GPa and expansion of 10% yielding a theoretical pressure of 167 MPa. Measured actuation pressures for paraffin-based designs yielded 27% [35] of the theoretical prediction, suggesting a gallium-based device can provide at least 480 MPa of pressure for robust actuation and pumping against back-pressure.

## 3. Fabrication

**Figure 4:**
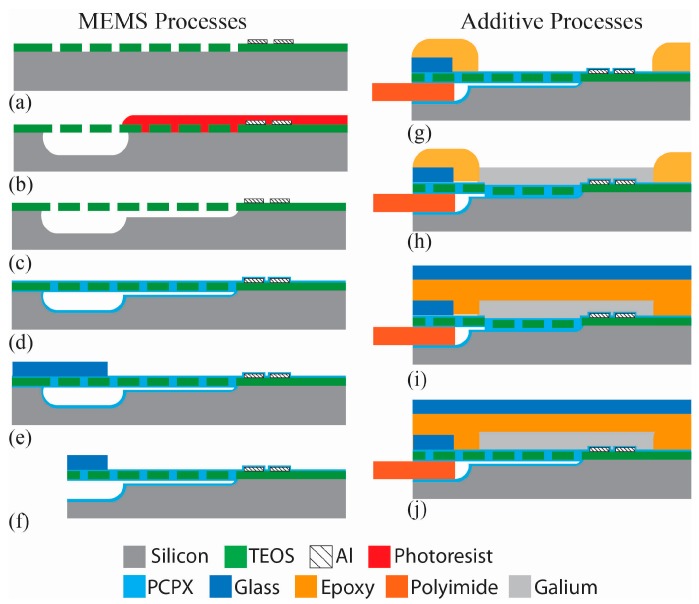
Micropump fabrication process with direct write integration over classic Micro Electro Mechanical System (MEMS) structures. (Not to scale) (**a**) 1 µm Tetraethyl Orthosilicate (TEOS) patterned to define channels and chambers with 0.3 µm Al heaters and contact pads; (**b**) Photoresist, XeF_2_ etch to 130 µm to define the interconnect channels; (**c**) Second XeF_2_ etch to create channels and chambers (15 µm depth) and to etch the interconnect channels to their final depth of 145 µm; (**d**) Parylene-C (PCPX) deposited (3 µm) to coat channels and form diaphragms; (**e**) Cover glass secured over interconnect area; (**f**) Wafer diced to reveal interconnect openings; (**g**) Epoxy resin printed to define plena, capillary tubing (140 µm OD) captured with 40 µm of Parylene-C; (**h**) Capillaries connected to vacuum with nanotight fittings to drawn down the diaphragms, gallium ingots (412 nL) placed in each of the four plena, melted, and re-solidified to hold the diaphragms before removing vacuum; (**i**) A Cover glass was manually coated with UV curable resin and lowered onto gallium filled plena; (**j**) When heated the gallium withdraws from the pump chamber pulling in fluid. The device can be encapsulated in Parylene-C to provide a biocompatible external surface.

Figure 4 shows a cross-sectional drawing of the pump fabrication process, which builds on integration technologies from [36]. Wafers were RCA cleaned and 0.5 μm of plasma-enhanced chemical vapor deposition (PECVD) Tetraethyl Orthosilicate (TEOS) SiO_2_ was deposited (Applied Materials P5000, 290 W, 68 s). The TEOS was patterned via a plasma etch (Drytek Quad, Ar: 35 sccm, CHF_3_: 65 sccm, O_2_: 5 sccm, 200 W, 70 mtorr) with a grid of 1-µm openings to define the fluid channel, interconnect, and diaphragm regions. A 0.3-µm film of aluminum was evaporated onto the surface in a CHA flash evaporator system. A contact mask was manually aligned to the patterned TEOS with exposure performed on a contact aligner (MA-150, Karl Suss America, Waterbury, VT, USA). Heaters and contacts were patterned in the aluminum with wet etching, Aluminum Etch 16-1-1-2 (Fujifilm Corporation, Tokyo, Japan).

The microfluidic channels, pump chambers, and interconnects to accept micro capillary tubing were formed with a two-step process that creates both shallow flow channels and deeper channels for the microfluidic interconnect. A film of photoresist was patterned to cover the shallow channel and chamber regions from an initial XeF_2_ etch process. The photoresist was then removed in an O_2_ plasma followed by a second XeF_2_ etch to form the shallow flow channels and pump chambers. This two-step process achieves different depths for the flow path (deep interconnects of 145 µm; shallow channels of 15 µm). The diaphragms were formed over the chambers by filling in the grid of 1-µm patterned openings in the TEOS film with a room temperature deposition of 3 µm of Parylene-C using a custom designed deposition tool. The thickness of the Parylene-C deposition was controlled by the amount of dimer placed in the evaporator at the start of the process, with one gram of dimer yielding 0.8 µm of polymer at a deposition pressure of 30 mtorr. This deposition simultaneously coats the microfluidic channels with biocompatible parylene while creating the deformable pump membranes [36]. Polyimide tape was applied to the resistor contact pads prior to deposition to keep these regions free of Parylene-C.

Microfluidic interconnects to polyimide microcapillary tubing were created as described in [37]. Briefly, a 0.17 mm cover glass (18 mm × 18 mm) was adhered to the chip along the interconnect edge to prevent resin from flowing into the microchannels during the direct write step and to provide a clean opening for the insertion of the capillary tubing. The wafer was diced with a wafer saw (K&S 780, Kulicke & Soffa Pte. Ltd., Singapore, Singapore) to expose the microchannel interface. Using a direct write system (nScrypt, Orlando, FL, USA) the actuator plenums were printed directly on top of the diaphragm device with UV curable resin to define the gallium wells. The resin was dispensed from a pneumatically controlled syringe (20.1 psi) through a 75 µm ID tip to control the width and height of lines defined in a tool path text file. The device was held onto the tool’s platen with vacuum and moved along the path at 5 mm/s. The resin gel (F-134, Art Clay World, Jacksonville, TX, USA) was cured under a 365 nm UV light for 10 min. Small diameter capillary tubing (140 µm OD) was inserted into the interface openings and sealed with a thick (40 µm) deposition of Parylene-C as described in [37]. Polyimide tape was applied the tops of the plena prior to deposition to keep these free of Parylene-C.

**Figure A1:**
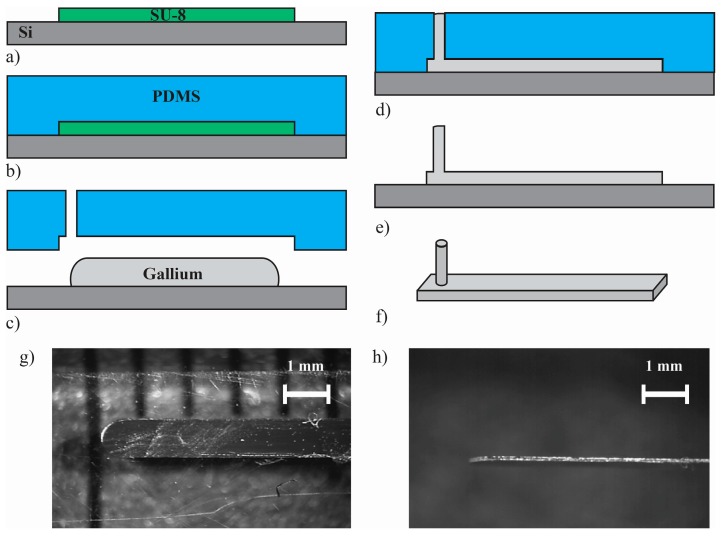
Images of micro-contact mold process. (**a**) SU-8 patterned lithographically on Si substrate; (**b**) PDMS poured over positive SU-8 mold, cured, then lifted free; (**c**) PDMS mold pressed against molten gallium on Si substrate; (**d**) Gallium was solidify by cooling with ice pack; (**e**) PDMS mold pulled off leaving gallium on substrate; (**f**) Free standing gallium ingot; (**g**) Top view of micromold-printed gallium ingot; (**h**) side view of gallium ingot.

To facilitate the precise measurement of the minute volume of Ga needed (412 nL of solidified Ga), lengths of long flat Ga ‘ingots’ were formed using a micro stamp-mold process using a polydimethylsiloxane (PDMS) mold. As shown in Appendix A, Figure A1, a positive mold was created using 105-μm thick SU-8 3010 photoresist (MicroChem Corp. Westborough, MA, USA) on a 100-mm silicon wafer. This positive mold was then used to cast the negative mold in PDMS, which had been processed under vacuum for 60 min to remove bubbles, followed by curing at 70 **°**C for 60 min. Individual stamps were razor cut with vent holes (1/2 mm OD) punched using a metal cylinder. Gallium was placed on a clean silicon wafer and melted on a hot plate at 100 **°**C. The stamp was pressed down on top of the molten gallium and held with a small weight. The wafer was then placed on an ice pack to speed cooling and solidification of the gallium micro-ingots, which were easily removed from the pliable PDMS mold. The resulting micro-printed ingots were 0.8 mm wide, 90 µm thick, and 20 mm long, providing a Ga volume of 72 nL per 1 mm length. Ingots were cut to a length of 5.7 mm with a razor blade under a 40× microscope, providing 412 ± 7.2 nL of Ga for placement in the pump actuation plenums. 

The capillary tubing was connected with Nanotight fittings (Upchurch, Scientific, Oak Harbor, WA, USA) to vacuum of less than 240 torr (providing a trans-diaphragm pressure of >10 psi) to hold the diaphragms in the down (closed) position in order to deposit the gallium. This ensures the diaphragms are actuated to the closed position by the solidified metal. Gallium micro ingots were placed in the plena and melted on a hot plate at 100 °C, such that they would conform to the shape of the actuated diaphragms while the pumps were under vacuum.

The device was removed from the hotplate and as the metal cooled it expanded and solidified, keeping the diaphragms pushed down as the vacuum was released. A second glass cover slip was coated with UV curable resin and affixed to the device to cover the actuation plena and seal in the gallium, providing a rigid cap for the actuation chambers. This resin was cured for 10 min under a 365 nm wavelength UV light.

## 4. Testing and Characterization Methods

Two characterization devices were designed, one to verify diaphragm deflection and quantify the required actuation pressure, and one for long-term diaphragm actuation testing.

### 4.1. Diaphragm Deflection

**Figure A2:**
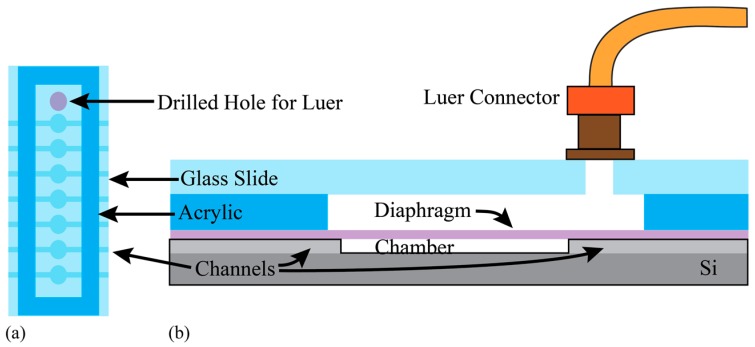
Illustration of cross-section of test fixture for testing diaphragm deflection. Visual inspection with Wyko interference microscope allows for correlation between actuation pressure and deflection at the center of the diaphragm for comparison to theory. (**a**) Top view showing pneumatic access, plenum, and underlying chamber; (**b**) Illustration of plenum/chamber arrangement, (pneumatic access shown out of position for clarity).

**Figure A3:**
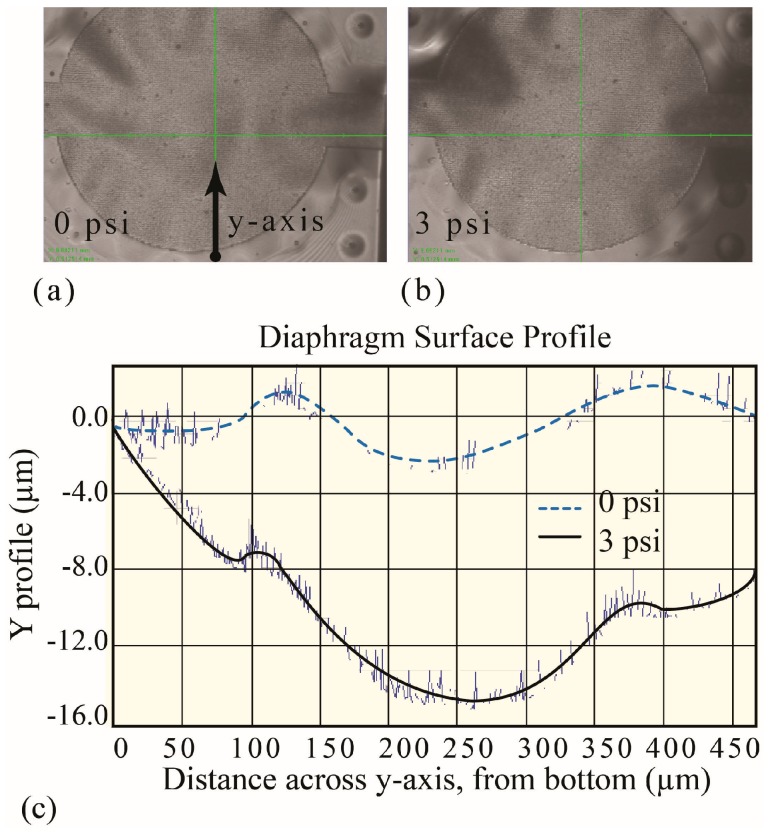
Membrane deflection images and graph. (**a**,**b**) From the Wyko interferometer of the pump diaphragm at rest, 0 psi, and at 3 psi; (**c**) Data from the Wyko showing the maximum deflection at the center of a 500-µm diameter diaphragm.

To characterize diaphragm deflection, a plastic plenum was fabricated from acrylic (1-mm thick) and adhered to a diaphragm chip using cyanoacrylate adhesive and covered with a glass cover slide as shown in Appendix A, Figure A2. The chip contained seven identical diaphragms each with an inlet and outlet channel with pneumatic access to the diaphragms provided through a nanoport fitting (Upchurch, Scientific, Oak Harbor, WA, USA) glued to the glass slide with a hole drilled through it. The stack of device/plenum/glass was viewed under a Wyko interferometric microscope (Veeco, Plainview, NY, USA) while pressure was applied to the plenum via compressed air. The Wyko allows for deflection measurements through the microscope slide as shown in Appendix A, Figure A3. An NPC-100 pressure sensor (GE Novasensor, Inc., Fremont, CA, USA) was used to record the pressure required to deflect the diaphragm.

### 4.2. Long-Term Survivability

**Figure 5:**
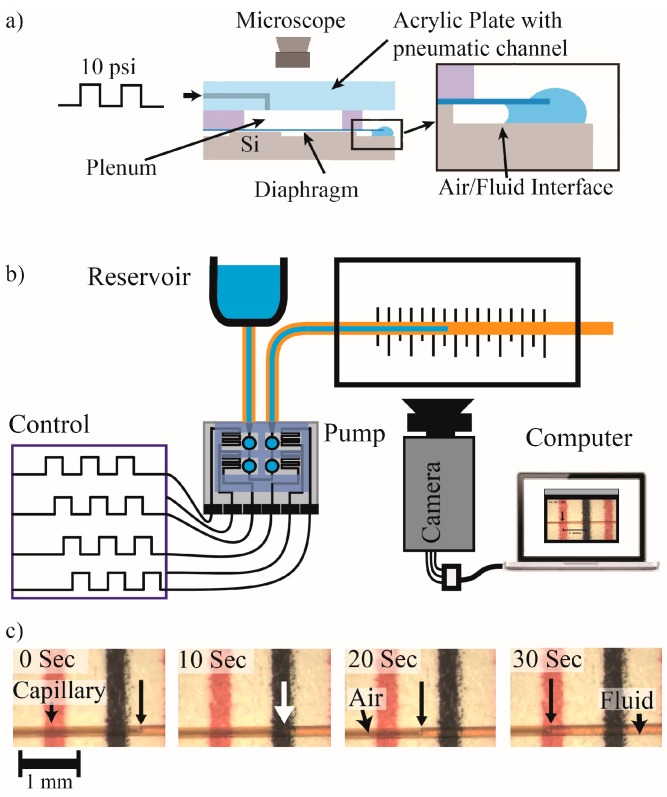
(**a**) Illustration of the long-term diaphragm deflection test set-up. Magnification shows a close up of the air/water interface examined on a weekly basis; (**b**) Set-up for gallium thermal actuation peristaltic pump characterization; (**c**) Photographic sequence of the output capillary over printed gradations for fluid movement measurement demonstrates 1.5 mm movement (14.8 nL) over 30 s for a pump rate of 29.6 nL/min.

Long-term testing was conducted on diaphragm chips described above with a plenum formed with a gasket and an acrylic plate as shown in Figure 5a. Compressed air was regulated to 10 psi and connected to a relay controlled by a triangle waveform from a wave generator (10 Vpp at 1 Hz). The solenoid passed the 10-psi air pulses to the plenum over the diaphragms and bled to atmosphere when turned off. Visual access through the acrylic plate allowed detection of the diaphragm deflection. One inlet/outlet side of the channels was filled with water forming an air/water interface at the distal end of the channels that could be observed during deflection to verify movement synchronized with the air pulses. To ensure the diaphragms were still intact throughout the duration of the long-term tests, steady pressure was periodically applied for 120 s to deflect the diaphragm and force air through any breached membranes. During this test the membranes and channels were inspected under a microscope to confirm no air leakage. This was done weekly and at the completion of the long term testing with no observed breach in the membranes or movement of the air/water interface, indicating fully intact diaphragms. The membranes were cycled through 26 million cycles, correlating to over 1.25 years of life for a pump averaging 100 nL/min continuous flow rate.

### 4.3. Micropump Characterization

The experimental setup for the gallium thermal actuation peristaltic pump is shown in Figure 5b. A camera recorded the fluid movement through the capillary tube as it was pumped from a reservoir (elevated syringe body) to the outlet capillary tubing. The volume of fluid pushed by each of the four chambers was determined experimentally by actuating and de-actuating through application of current. 32 mA was supplied to each heater, raising the Gallium temperature to 306 K (three degrees above the melting point) as estimated with COMSOL simulations as described below. The fluid/air interface within the microcapillary tubing was observed under a microscope, with movement distance correlated to volume (Volume/Length = 9.85 nL/mm).

To measure the pumping speed, the chambers were actuated peristaltically with a control circuit designed using 555 timers, flip-flops, and logic circuits to regulate the power applied to the actuation heaters. The power was supplied with four current sources (32 mA) each using two NPN transistors (2N3904). The current sources are turned on with a signal generated by the timers and flip-flops with one timer controlling the frequency of the actuation sequence and the other switching the control signal on and off within the envelope of the actuation sequence.

## 5. Results and Discussion

**Figure 6:**
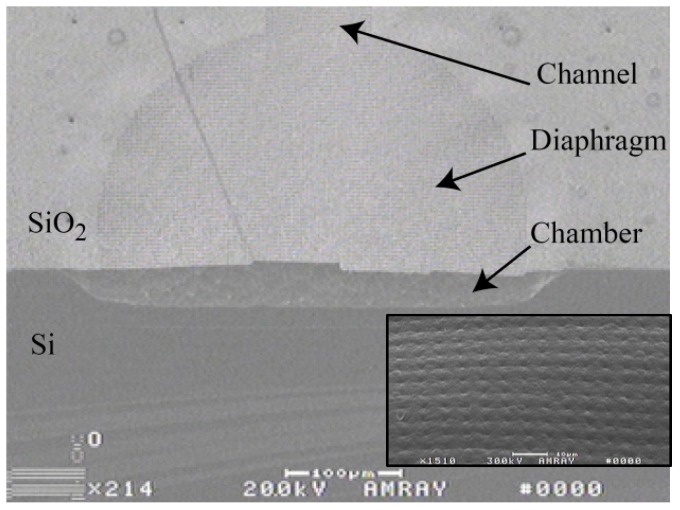
SEM of *in-situ* formed Parylene-C encapsulated TEOS diaphragm over a micropump chamber. *Inset*: Close-up of diaphragm visualizing dimples in the Parylene-C surface corresponding to the etched holes in the underlying TEOS framework.

**Figure 7:**
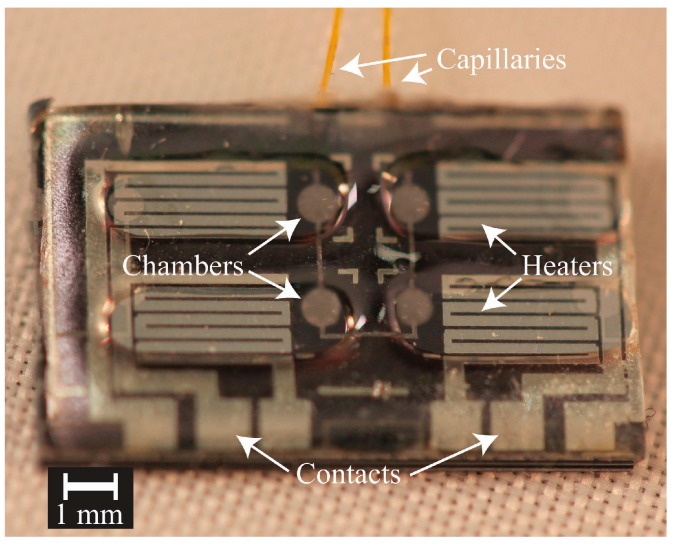
Photograph of pump with plena and capillary tubing. Visible are the interconnected capillary tubes, heaters, diaphragm chambers, plena, and heater contact pads. Heater miniaturization was limited by resolution of the printed transparency masks used in this work.

As shown in Figure 6, a TEOS grid is formed over the channels and chambers etched into the silicon wafer forming the peristaltic pump structure; the flexible PCPX, filled in the grid and formed a contiguous diaphragm. A thin film of Parylene-C was seen to coat the channels and chambers below the diaphragms [23], providing a biocompatible flow path through the pump. Figure 7 shows the device prior to Galium placement. The underlying microfluidics and heaters were successfully formed using MEMS techniques with the printed epoxy defining the plena above. The small diameter capillary tubing (140 µm OD) was captured in place via parylene deposition as detailed in [37].

The diaphragm deflection tests on devices with chamber diameters of 500 μm, a depth of 15 μm, and a diaphragm of 1 μm TEOS and 4.5 μm PCPX showed that the diaphragms were fully deflecting to contact the floor of the etched chamber below. Measurement of the required deflection pressure of 3 psi shows modest agreement with the calculated value of 3.3 psi as shown in Appendix A. To broaden the potential actuation mechanisms for these diaphragm membranes, additional devices were fabricated with a larger diameter chamber (800 μm) and slightly thinner PCPX (3 μm) to reduce the required actuation force. While the gallium provides more than adequate actuation pressure, the more compliant diaphragms may enable adaptation to other actuation mechanisms with lower force capabilities such as electro-osmosis, electrolysis, and piezoelectric [37].

In the long-term testing, seven diaphragms (500 μm diameter, 1 μm TEOS, and 4.5 μm PCPX) were actuated for 307 days with over 26 million (26,524,800) actuations with no failures. This is the equivalent of over 185 million diaphragm actuations without a single failure. These results suggest the composite TEOS/Parylene membranes provide a robust interface between the actuator and the pumped fluid.

**Figure 8:**
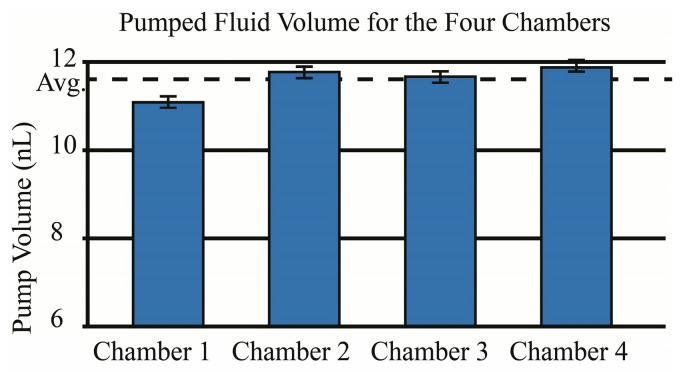
Volume of fluid pumped for the four pump chambers. *n* = 16, Average = 11.1 nL, σ = 0.35 nL.

Pump actuation tests on devices with chambers 800 μm diameter, 15 μm deep reveal a consistent volume pumped per cycle of 11.1 ± 0.35 nL as shown in Figure 8. The pump fabrication process results in a volume pumped per cycle that is dependent on the gallium volume and associated expansion. The pump membrane is able to deflect upward and downward, resulting in the capability for greater volumes than those defined by the chamber depth and diameter alone. The average pumped volume is 0.9 nL (7.5%) lower than designed which may be due to three sources of error. The pumped fluid is controlled by the dimensional change of the gallium, which is directly correlated to the Ga volume. The cutting error of the gallium was estimated to be ±7.2 nL; this would account for a pumped volume error of only ±0.01 nL. Another potential source of error is over heating of the gallium past the melting point due to open-loop heater control. COMSOL simulations of the pump structure suggests the Ga will reach temperatures of 306 K, three degrees higher than the melting point of pure Ga. The melted gallium will re-expand after the initial melted state is reached. The 0.9 nL reduction in pumped volume corresponds to a reduction in Ga density of 6.075 g/cm^3^ and a temperature overshoot of 20 **°**C, much higher than estimated with COMSOL so this is unlikely to be the source of error. The last potential source of error is from the integration process when the molten gallium is placed over the diaphragms as they are being held down with a vacuum. Deflection differences as small as 1.8 µm could explain the 0.9 nL volume error. For the future, positive pressure deflection during gallium integration may provide more robust membrane deflection and reduce this relatively small volume error. 

**Figure 9:**
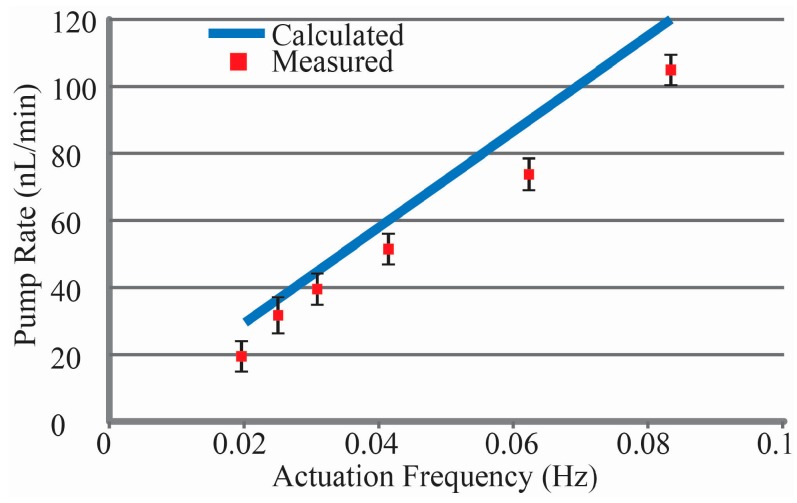
Pump rate *vs.* actuation frequency for the micropump compared to theoretical rates based upon chamber volume design (*n* = 3). Lower flow rates are possible with duty cycle control, while higher rates can be achieved with larger chambers and scaling of the Ga.

Flow rates were measured as described above for actuation frequencies from 0.02 to 0.083 Hz as shown in Figure 9, with pump rates between 18 and 104 nL/min achieved at an activation power of 10.1 mW and a Gallium temperature of 306 K. This four chamber peristaltic pump delivers two pump chamber volumes (e.g., 22.2 nL) every period, yielding an efficiency of 11 mJ/nL. These results compare favorably to phase change actuation micropumps operating in the laminar flow regime where efficiencies of 48 mJ/nL [38] have been reported. However, this Ga-based actuator offers additional efficiency benefits compared to traditional normally open micropumps for complex pump regiments and very low flow rate applications. Both require the pump to be off or paused for periods of time; the Ga-actuator closes off the flow channel when power is removed, eliminating the need to power an active valve. While the lowest pump rate presented was 18 nL/min, lower rates can be achieved by cyclic pausing of the pump with all chambers closed. Achieving pump rates higher than 104 nL/min can be achieved through increasing the actuation current to shorten melt times, or by increasing the size of the pump chambers and associated Ga.

The melting point of pure Gallium is 303 K (30 **°**C) and required a temperature rise of 10 **°**C from room temperature of 293 K for our pump testing. There are two main issues associated with this temperature range: a large shift in temperature requires power, and the melting point is below body temperature (37 **°**C). The melting point needs to be optimized for use in implantable systems using a gallium-indium (Ga-In) alloy; An alloy of 23% atomic weight In will provide an increase in the melting point from 30 to 40 **°**C [39]. Since the melting point of indium is relatively low (156 **°**C) and the boiling point of Ga is over 2200 **°**C, the alloy can be formed without significant loss of either metal. An alloy with a transition temperature tuned to be just a few degrees above body temperature will reduce power requirements for heating and improve efficiency. It is expected that reducing the required temperature rise from 10 to 3 **°**C could yield pump efficiencies of 9.2 mJ/nL.

COMSOL simulations of the pump structure reveals overshoot in target gallium temperature due to the constant application of power even after the gallium had reached its melting temperature. Closed loop control would enable attenuation of power as the temperature approaches the melting point to eliminate this overshoot. The COMSOL model suggests power reductions down to 6.3 mW are possible, providing a theoretical pump efficiency of 6.8 mJ/nL. Closed-loop control would require integration of temperature sensing within the plenum, which could be achieved with thermopiles as described by [40,41,42]. A recent review by Ogden on miniature paraffin phase change actuators, valves and pumps [43] provides insight into the power and associated flow rates for a range of system designs. The most efficient of the pumps reviewed [44], was shown to have flow rates as low as 80 nL/min but at an estimated efficiency of 18 mJ/nL, roughly three times less efficient than the Ga pump presented here.

The pump rate is easily controlled via the frequency of the control circuit; this pump demonstrated a large range of pump rates (18 to 104 nL/min). For much lower flow rates, smaller chambers could be used, along with smaller gallium plenums. The chambers used here were 800 µm in diameter and 15 µm deep. Chambers as small as 400 µm in diameter and 5 µm deep to as large as 1500 µm in diameter and 20 µm deep have been successfully fabricated. This range of demonstrated chamber sizes enables scaling of the flow rate from 1.5 to 0.5 μL/min with the described MEMS technology. Multiple flow channels could be employed to magnify the flow rates. For example two pumps in parallel could pump up to 1 µL/min and if the chambers of each pump were arranged linearly, the pumps could share electronics reducing the device’s complexity. To increase the flow rate beyond µL/min flow rates, other chamber and diaphragm technologies could be integrated with the gallium actuators. Molded PDMS chambers and diaphragms, used for thermodynamic actuation, can be fabricated with diaphragm diameters >5 mm [45].

The pseudo-conformal nature of the Parylene-C deposition process ensures that all flow paths are coated with a non-reacting, non-water-adsorbing, biocompatible material [31,37]. This allows the pumping of fluids commonly used for infusions, including pharmaceuticals and biologic agents without altering the fluids or the pump. Even with the biocompatibility of the Parylene-C encapsulation of the device, concerns of potential toxicity leakage of materials must be addressed. The toxicity of Ga, In, and Ga-In alloys in humans is not well studied and the Material Safety Data Sheet for Ga-In only presents limits on inhalation (<0.1 mg/m^3^) [46]. Small animal studies in rats with intratracheal administration of Ga, at doses of 24, 48, and 96 mg/kg, revealed temporary toxicity effects (<18 days) for highest dose only, but that no effects were seen for lower doses [47]. The oral median lethal dose (LD50) of Ga in mice and rats is more than 15 g/kg, and the threshold for acute effects (TAE) is 7 g/kg [48]. The oral LD50 of indium is 4.2 g/kg for rats [49]. The presented micropump designed for small rodents would contain 0.22 mg of Ga and 0.064 mg of In, which for a 10 g mouse is the equivalent of 22 and 6.4 mg/kg respectively. The Ga is two orders of magnitude lower than the TAE and three orders of magnitude lower than the LD50. The In is three orders of magnitude lower than the TAE and four orders of magnitude lower than the LD50. While additional work is required to evaluate potential toxicity of leakage of the Ga-In alloy from a damaged micropump, present literature suggests the small volumes involved may have minimal impact, even in the smallest animal models.

## 6. Conclusions

A planar micropump has been presented that is low volume, low power, controllable, with a novel hybrid fabrication process combining traditional MEMS with direct write methods. The pump will work with fluids commonly used for lab-on-a-chip applications and in biological infusions in animal models with tuning of the actuation temperature via Ga-In alloys. A pump smaller than 100 mm^3^ was fabricated with demonstrated pumping between 18 and 104 µL/min linearly proportional to actuation frequency, and with a single actuation volume of 11 nL. A key benefit of the gallium phase-change actuation is blocked flow in the power-off state, enabling isolation of the drug reservoir from the delivery target without consuming energy. An efficiency of 11 mJ/nL was demonstrated, with theoretical improvement to less than 7 mJ/nL possible with the addition of closed-loop control and tuning the melting point of the gallium through alloying with indium. The presented pump and associated fabrication technologies will play an important role in future drug therapy research, especially where infusions to small biological structures in animal models and lab-on-a-chip applications require ultra-low flow rates.

## Figures and Tables

**Figure A4:**
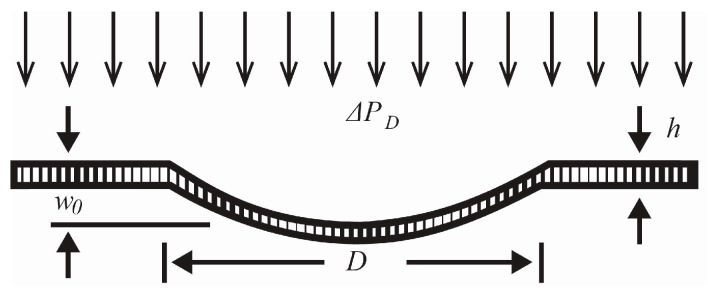
Diagram of parameters for diaphragm deflection. Large deflection (*w*_0_ > *h*) of diaphragms of diameter *D* under trans-diaphragm pressure Δ*P_D_*.

## Abbreviations

The following abbreviations are used in this manuscript:

PECVD	Plasma-Enhanced Chemical Vapor Deposition
TEOS	Tetraethyl Orthosilicate
µTAS	Micro Total Analysis Systems
*Re*	Reynolds number
PCPX	Parylene-C
PDMS	Polydimethylsiloxane
MEMS	Micro Electro Mechanical System
UV	Ultra Violet
OD	Outer Diameter
ID	Inner Diameter
NIDCD	National Institute for Deafness and Communication Disorders
RIT	Rochester Institute of Technology

## Appendix A

The fabrication process of gallium micro-ingots is shown in Figure A1. A positive mold is patterned lithographically on top of Si substrate with the SU-8 3010 (MicroChem Corp., Westborough, MA, USA). A negative mold is made with PDMS from this positive mold. The negative mold, prepared with release agent (Micro-90), is pressed upon molten Gallium, having been placed on a Si substrate and melted on a hot plate (50 **°**C). The gallium was allowed to solidify by removing from the hot plate and cooled with ice pack. The PDMS mold was pulled off leaving the gallium ingot on the substrate. The freestanding gallium ingot was measured and cleaved to provide a precise volume of solid gallium. The low aspect ratio of the ingot facilitates ease of cutting with razor blade to precise lengths.

The stack of device/plenum/glass (as shown in Figure A2) was viewed under a Wyko interferometric microscope (Veeco, Plainview, NY, USA) while pressure was applied to the plenum. Since the diaphragm is under one layer of glass, the interferometric microscope requires a similar sample of glass in the optical path in order for the interference to produce results, so a small chip, ~20 mm^2^, of a glass slide was affixed to a removable aperture and placed in the Wyko. The Wyko allows for deflection measurements through the microscope slide. An NPC-100 pressure sensor (GE Novasensor, Inc., Fremont, CA, USA) was used to record the pressure required to deflect the diaphragm. Figure A3 shows images from the Wyko of a diaphragm in the relaxed state at, Δ*P_D_* = 0 psi and deflected downward 15 µm at the center of the diaphragm.

(A1) $$w_{0}=\frac{\Delta P_{D}a^{4}}{64D_{f}}\frac{1}{1+0.488\frac{w_{0}^{2}}{h^{2}}}$$

(A2) $$D_{f}=\frac{Dh^{3}}{12\left(1-v^{2}\right)}$$

An actuation pressure of 3 psi was needed to fully deflect a 500-µm diameter diaphragm of 1-µm TEOS, and 4.5-µm Parylene-C. Using E-1 and E-2, from [50] (as shown in Figure A4), and the superposition of two diaphragms (one of TEOS and one of Parylene-C) gives an actuation pressure of 3.3 psi for a 15-µm deflection, which is an error of 10%. The approximation is performed by modeling the pressure required to deflect a 1-µm thick TEOS diaphragm and a 4.5-µm thick Parylene-C diaphragm separately, and adding the two pressures using an average thickness, where the average thickness is equal to the film thickness times the percent coverage (75%). Potential sources of error include non-uniformity in film thicknesses, and the non-planarity of the composite film.
